# Reply to the letter to the editor concerning the article “clinical outcomes of chondroblastoma treated using synthetic bone substitute: risk factors for developing radiographic joint degeneration”

**DOI:** 10.1186/s12957-021-02285-4

**Published:** 2021-06-12

**Authors:** Hidetatsu Outani, Shigeki Kakunaga, Yoshinori Imura, Takafumi Ueda

**Affiliations:** 1 Department of Orthopaedic Surgery, Osaka University Graduate School of Medicine, 2-2, Yamadaoka, Suita, Osaka, 565-0871 Japan; 2 Department of Orthopaedic Surgery, National Hospital Organization Osaka National Hospital, 2-1-14 Hoenzaka, Chuo-ku, Osaka, 540-0006 Japan; 3 Department of Orthopaedic Surgery, Kodama Hospital, 1-3-2 Gotenyama, Takarazuka, Hyougo 665-0841 Japan

To the Editor,

Thank you for providing us the opportunity to respond to the letter to the editor by Bo-Wen Zheng et al. We thank them for their interest in our study [1]. The main purpose of this study is to evaluate the safety and effects of using synthetic bone substitute (SBS) on adjacent-joint for filling the cavity after curettage of chondroblastoma, which often develops around a joint [2]. Due to the rarity of this disease, we were able to collect only 40 patients for analyses and we agree with the weak statistical power and lack of a control group as they mentioned. Therefore, we acknowledged these limitations in the last part of the discussion. In addition, in the discussion, we compared the incidence of radiographic degeneration in our cohort to that of previous case series and found low incidence in our cohort [3]. Accordingly, we concluded SBS can be used safely around a joint. They also suggested prospective controlled studies with large samples. However, considering the rarity of this disease, conducting a randomized clinical trial with well-controlled tumor background seems to be difficult. Regarding the cut-off point of age, we used 14 years because younger age (less than 14 years) was mentioned as a risk factor of local recurrence [4]. We again thank Bo-Wen Zhen et al. for their interest in our study, and we hope our small retrospective case series would be helpful when surgeon plans to use SBS for surgery of chondroblastoma.
